# Borneo herbal plant extracts as a natural medication for prophylaxis and treatment of *Aeromonas hydrophila* and *Pseudomonas fluorescens* infection in tilapia ( *Oreochromis niloticus*)

**DOI:** 10.12688/f1000research.16902.2

**Published:** 2019-02-13

**Authors:** Esti Handayani Hardi, Rudy Agung Nugroho, Irawan Wijaya Kusuma, Wiwin Suwinarti, Agung Sudaryono, Rita Rostika

**Affiliations:** 1 Microbiology Laboratory, Department of Aquaculture, Faculty of Fisheries and Marine Science, Mulawarman University, Samarinda, East Kalimantan, 75123, Indonesia; 2 Animal Physiology, Development and Molecular Laboratory, Department of Biology, Faculty of Mathematics and Natural Sciences, Mulawarman University, Samarinda, East Kalimantan, 75123, Indonesia; 3 Laboratory of Forest Product Chemistry, Faculty of Forestry, Mulawarman University, Samarinda, East Kalimantan, 75123, Indonesia; 4 Department of Aquaculture, Faculty of Fisheries and Marine Science, Diponegoro University, Semarang, Central Java, 50275, Indonesia; 5 Department of Fisheries, Faculty of Fisheries and Marine Science, Padjajaran University, Bandung, West Java, 40600, Indonesia

**Keywords:** Imunomodulator, Concoction, Aeromonas hydrophila, Pseudomonas fluorescens, Prophylaxis

## Abstract

**Background:** The combination of some plant extracts to prevent and treat bacterial infections is gaining momentum, because of effectiveness against certain bacteria. This study aims to describe the antibacterial and immunostimulant abilities of *Boesenbergia pandurata *(BP), *Solanum ferox *(SF) and *Zingiber Zerumbet* (ZZ) plant extracts to treat and prevent *Aeromonas hydrophila* and *Pseudomonas fluorescens* infection on Tilapia ( *Oreochromis niloticus*).

**Methods:** Tilapia (initial weight 15±2 g) were injected intramuscularly (0.1 ml/fish) with a combination of *A. hydrophila* and *P. fluorescens* at a density of 1×10 ^5^ CFU ml ^-1^ of each bacteria. Treatment trials were performed at day 7 post-injection with each combined extract, while the prevention trial was performed by including the combined extract into the commercial diet for six and seven days prior to injection. Various extract combinations were 60 mg SF extract/kg feed with 40 mg ZZ/kg feed (SF60/ZZ40), SF50/ZZ50, BP90/SF10, and BP50/SF50. Haemato-immunological parameters were performed for four weeks.

**Results:** In prevention trials, tilapia fed SF50/ZZ50 showed a significant increase of white and red blood cells. Similarly, significantly increased haematocrit was found in tilapia fed SF50/ZZ50 in the treatment trial but not in the prevention trial. In both trials, haemoglobin of tilapia was not affected by any combined extracts but decreased the number of bacteria. Phagocytic index, respiratory burst, lysozyme activity and survival rate of fish fed combined extracts were found significantly higher than controls. The amount of pathogenic bacteria in fish fed combined extracts was lower than the control at week 4 ( *P<0.05*). In both trials The percentage of survival rate and relative percent survival of tilapia fed SF 50/ZZ 50, showed the optimum results compared to the other combinations.

**Conclusions:** The combined extract in feed, especially SF50/ZZ50 has a positive effect on the tilapia's innate immune system of tilapia to treat and prevent bacterial infections.

## Introduction

Tilapia ( *Oreochromis niloticus*) is one of the most widely cultivated fish species in Indonesia. Tilapia is a freshwater fish that can be easily cultivated ^1^. According to Pridgeon ^2^ and Harikrishnan *et al*. ^3^, freshwater fish culture is inseparable from bacterial infections which are caused by motile *Aeromonas* septicaemia, furunculosis, edwardsiellosis and *Aeromonas hydrophila*. Further, *Aeromonas* species have been identified as major causative bacteria and a serious pathogen in fish ^4, 5^. In Indonesia, particularly East Kalimantan, infection of *A. hydrophila* and *Pseudomonas fluorescens* in fish results in high mortality rates of up to 60–80%. In fish, both of these bacteria cause stresses, exophthalmia, ulcers, and watery-looking organs, particularly gallbladder rupture ^6– 8^. In addition, combined bacterial infection in fish is also common, such as infections found in tilapia caused by *Salmonella agalactiae* and *A. hydrophila* ^9, 10^.

To reduce high mortalities of cultured fish, aquaculturists and researchers use antibiotics to prevent and treat infection. Nevertheless, due to concerns for maintaining eco-friendly environments, the application of antibiotics should be avoided, because they may enhance antibiotic-resistant pathogens, increase the accumulation of drugs in fish tissue and trigger immunosuppression ^11^. Methods of controlling these infections should be developed as soon as possible because the pathogen disease type has significantly increased ^12^, while the type of pathogen that leads to edema in the cultivation area still cannot be overcome. One of the effective and safe methods for disease control in aquaculture is by improving the defence system of the fish through the provision of natural immunostimulants ^13^, through the use of several plant extracts.

Recently, the popularity of plant extracts as natural immunostimulant is gaining in demand and importance in medical purposes. Various plant extracts, such as Indian almond leaves ( *Terminalia catappa*), oats ( *Avena sativa*), oyster mushroom ( *Pleurotus ostreatus*), nettle ( *Urtica dioica*), sea grass ( *Cymodocea serrulata*) and beetroot ( *Beta vulgaris*) have been used as alternatives to antibiotics ^5, 14– 16^. Plant extracts also contain levamisole ^13^ and saponin ^17^ which can enhance the work of nonspecific immune systems and increase the activation of phagocytosis ^14^. Plant extracts could optimize the fish blood function, by enhancing the number of white blood cell to prevent the bacteria ^5^. Further, the plant extracts of *Boesenbergia pandurata* (BP) and *Zingiber zerumbet* (ZZ) from East Kalimantan have *in vitro* and *in vivo* antibacterial activity against *A. hydrophila* bacteria, while *Solanum ferox* (SF) has been found to be an antibacterial agent for *P. fluorescens* bacteria. Similarly, for the prevention and treatment of bacterial infections in tilapia, BP and ZZ are also effective for treating *A. hydrophila* and *P. fluorescens* infection ^8, 18^.

The incorporation of some extracts for the prevention and treatment of bacterial infections is likely to increase the effectiveness because some materials can work synergistically, so that the infection of both bacteria in the fish body can be controlled optimally. However, research regarding the combination of plant extracts to treat and prevent bacterial infection is limited. This study therefore aims to determine the effectiveness of the combination of three extracts (BP, ZZ and SF) to prevent and treat bacterial infections of *A. hydrophila* and *P. fluorescens* in tilapia.

## Methods

### Fish and bacteria

In total, 450 Tilapias (Initial weight 15 ± 2 g, age ±2.5 months, random sex) were obtained from Teluk Dalam Village in Tenggarong Seberang, Kutai Kartanegara, Indonesia. The fish were randomly distributed and assigned into five aquariums in triplicates, representing four treatments and one control. The fish were kept in the laboratory for two weeks for acclimatization in the aquarium (60×40×30 cm). Each aquarium was filled with 60 l of freshwater and the water was changed by as much as 50% every 2 days to remove remaining faeces and inedible feed. The average temperature of the water was 27°C. The feed given in the acclimation phase was a commercial feed (PT Rama Jaya Mahakam, Kutai Kartanegara East Kalimantan-Supplier, floating pellets, containing 31–33% protein and 4–6% fat) at a rate of 5% of the body weight of the fish per day. The bacteria used for the challenge test were *A. hydrophila* (EA-01) and *P. fluorescens* (EP-01), which was provided from the Aquatic Microbiology Laboratory, Faculty of Fisheries and Marine Sciences, Mulawarman University, Indonesia. To bring about bacterial challenge, a combination of bacteria at density of 10 ^5^ CFU ml ^-1^ of each bacteria was used. Each fish was injected intramuscularly with 0.1 ml of the suspension of the bacteria.

### Plant and chemical materials

The plant materials, BP, SF and ZZ, were collected from a traditional market in Samarinda City, East Kalimantan, Indonesia. The plants were cleaned, cut and dried at 40°C for 48 hours in the oven, finely powdered and stored at -4°C for the further extraction stage. Ethanol solution (95%) was used to extract the plant materials, following a method described by Limsuwan & Voravuthikunchai ^19^. All chemicals used in this research was obtained from commercial sources (Sigma Aldrich, Inc. USA).

### Experimental design and challenge test

This treatment and prevention trials were carried out for 28 days. The treatment experiments were conducted with five combination treatments with the following stages: tilapia (average initial weight 15 ± 2 g, n = 30 fish per group, random sex) were injected intramuscularly (0.1 ml) with a mixture of *A. hydrophila* and *P. fluorescens* bacteria, each bacteria at density10 ^5^ CFU ml ^-1^. At day 7 after injection, the fish were fed with feed combined with extract as follows (mg per kg feed): P1, 60 mg SF extract/kg feed with 40 mg ZZ extract/kg feed (SF60/ZZ40); P2, SF50/ZZ50; P3, BP90/SF10; P4, BP50/SF50; and P5, fed with no additional extract (control). All fish were fed twice a day *ad satiation*. The remaining feed was siphoned out before the next feeding.

Meanwhile, the prevention trial was performed by providing the same feeding combination and procedure for 6 days prior to intramuscular injection of the fish with 0.1 ml of mixed bacteria at day 7. After injection, feeding combination was continued until the 4 ^th^ week. Haematological and immunological parameters were measured every week after the injection with bacteria until week 4.

### Haematology and phagocytic index

At days 14, 21 and 28 following bacterial challenge, haematological profiles of fish (n=3 per treatment group) were observed. Fish were anesthetized using 50 mg l ^-1^ MS 222 (Sigma Aldrich, USA) / dm ^3^ water. The fish blood was taken through the caudal vein, using a 1 ml syringe rinsed with 10% trisodium citrate anticoagulant (fish were kept alive after blood withdrawal). Total red blood cells (RBC) (10 ^6^ per mm ^3^) and white blood cells (WBC) (10 ^3^ / mm ^3^) were determined manually using an improved Neubauer counting chamber. The number of WBC was calculated using the method of Blaxhall and Daisley ^20^. Haemoglobin (Hb) was measured spectrophotometrically at 540 nm using the cyanmethemoglobin method ^17^. The haematocrit (Htc %) was counted using the microcentrifuge and heparinized was used as a standard solution. Meanwhile, phagocytic index was determined using a modification of previous methods by adding Turk solution into suspension of fish blood and bacteria to remove red blood cells. Thus, the number of white blood cells can be easily counted ^20– 22^.

### Respiratory burst and lysozyme activity

Respiratory burst activity test was performed using nitro blue tetrazolium (NBT) reagent, using the method outlined by Secombes and Olivier ^23^. Meanwhile, lysozyme activity was performed using a microtiter plate ELISA reader at wavelength of 520 nm, following the method described by Soltani and Pourgholam ^24^.

### Total Plate Count

To perform the total plate count (TPC), a blood sample of each fish from each group was homogenized and diluted in physiological saline solution of 0.85%. The dilutions were then transferred to bacterial counts. The TPC was conducted following the method of Turkogfu *et al.* ^25^.

### Disease resistance

Both *A. hydrophila* and *P. fluorescens* (the pathogenic bacteria) were used for challenge testing (n = 10 fish per aquarium, in triplicates per group). The survival rate (SR) and relative percent survival (RPS) of the fish were recorded on a daily basis for 4 weeks ^26^.

### Statistical analysis

Results are expressed as means ± standard error (SE) and the data were analysed using SPSS version 22 (SPSS, Inc., USA). The data of WBC, RBC, haematocrit, Hb, TPC, phagocytic index, respiratory burst and lysozyme activity were subjected to ANOVA, followed by Duncan’s post hoc test to evaluate significant differences among the groups of treatments. The percentage of fish survival were arcsine-transformed. All tests were significant at *P* < 0.05.

## Results

### Haematological profile

The present results revealed that the total WBC count of tilapia in the treatment and prevention trials were significantly increased ( *P*<0.05) from weeks 2–4 post-administration with combined extracts. The highest increase of WBC was found in tilapia fed with SF50/ZZ50. Similarly, total RBC and haematocrit of tilapia fed SF50/ZZ50 in the treatment trial showed a significant increase after week 2, while tilapia fed SF60/ZZ40 in the prevention trial led to a positively enhanced result from weeks 2–4. Further, haemoglobin of fish both in treatment and prevention trials were not affected by any various combination of extracts ( Table 1).

**Table 1.** Hematological profile of Tilapia ( *Oreochromis niloticus*) fed different extract combination in treatment and prevention trials.

Variables	Trials	groups	Weeks	
			2	4
WBC (10 ^4^ cell/mm ^3^)	Treatment	A	1.68±0.1 ^a^	2.07±0.2 ^b^
		B	3.60±0.1 ^b^	8.85±0.2 ^c^
		C	1.88±0.5 ^a^	2.10±0.1 ^a^
		D	1.98±0.5 ^a^	2.20±0.1 ^b^
	E	1.35±0.2 ^a^	1.34±0.1 ^a^
	Prevention	A	1.85±0.15 ^a^	2.4±0.5 ^b^
		B	3.9±0.2 ^b^	7.96±0.2 ^c^
		C	2.0±0.2 ^a^	2.4±0.3 ^b^
		D	2.0±0.3 ^a^	2.5±0.1 ^b^
		E	1.4±0.5 ^a^	1.3±0.1 ^a^
RBC (10 ^6^cell/mm ^3^)	Treatment	A	5.4±0.3 ^a^	4,2±0.1 ^b^
		B	7.7±0.2 ^b^	8.8±0.2 ^c^
		C	6.3±0.1 ^b^	7,2±0.2 ^a^
		D	6.8±0.2 ^a^	7.0±0.1 ^b^
		E	2.7±0.1 ^a^	2.7±0.1 ^a^
	Prevention	A	6.42±0.25 ^a^	6.0±0.5 ^b^
		B	6.97±0.3 ^a^	7.0±0.2 ^a^
		C	6.23±0.1 ^a^	5.53±0.2 ^a^
		D	5.67±0.1 ^a^	7.0±0.1 ^a^
		E	2.7±0.2 ^a^	2.47±0.1 ^a^
Hematocrit (%)	Treatment	A	27±0.1 ^a^	34±0.1 ^b^
		B	27±0.2 ^b^	36±0.2 ^c^
		C	27±0.2 ^a^	31±0.2 ^a^
		D	27±0.2 ^a^	30±0.2 ^a^
		E	15±0.3 ^a^	15±0.2 ^a^
	Prevention	A	27±0.1 ^a^	27.7±0.1 ^a^
		B	27±0.2 ^a^	34±0.1 ^a^
		C	27±0.1 ^a^	31.5±0.1 ^a^
		D	27±0.1 ^a^	30±0.2 ^a^
		E	14.7±0.2 ^a^	15±0.1 ^a^
Hemoglobin (g dL ^-1^)	Treatment	A	10±0.3 ^a^	10±0.1 ^a^
		B	10±0.3 ^a^	10±0.1 ^a^
		C	10±0.2 ^a^	10±0.1 ^a^
		D	8±0.2 ^a^	10±0.1 ^a^
		E	8±0.1 ^a^	6±0.2 ^a^
	Prevention	A	10±0.1 ^a^	8±0.2 ^a^
		B	10±0.1 ^a^	10±0.1 ^a^
		C	10±0.1 ^a^	8.3±0.1 ^a^
		D	8±0.1 ^a^	8±0.2 ^a^
		E	8±0.1 ^a^	4±0.1 ^a^

Note: Mean±standard deviation followed by different superscript letters (a,b,c) in the same column in each variable and each treatment or prevention trial showed significantly different at *P<0.05*. WBC = White blood cell, RBC = Red blood cell, A = SF60/ZZ40, B = SF50/ZZ50, C = BP90/SF10, D = BP50/SF50, E = No extract addition. Extract ratio is in mg per kg feed. BP = *Boesenbergia pandurata*), SF = *Solanum ferox*, and ZZ = *Zingiber Zerumbet*. In treatment and prevention trials, fish was infected with *Aeromonas hydrophila* and *Pseudomonas fluorescence.*

### Phagocytic index

**Figure 1.**
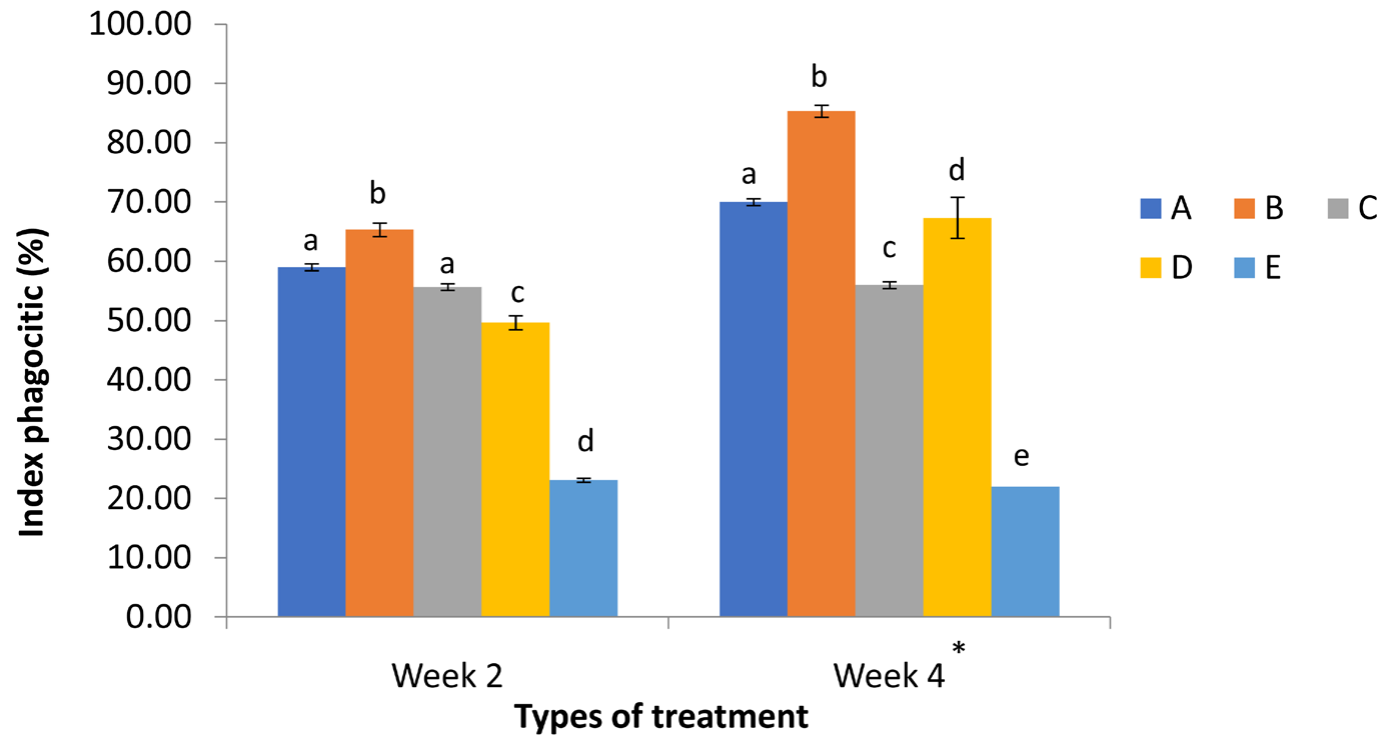
Phagocytic index (%) of Tilapia ( *Oreochromis niloticus*) fed different extract combination in treatment trials. BP = *Boesenbergia pandurata*), SF = *Solanum ferox*, and ZZ = *Zingiber Zerumbet*. In treatment trials, fish was infected with *Aeromonas hydrophila* and *Pseudomonas fluorescence.* Infected fish fed various combination and ratio of extract, namely: A = SF60/ZZ40, B = SF50/ZZ50, C = BP90/SF10, D = BP50/SF50, E = No extract addition. Extract ratio is in mg kg ^-1^ feed. * = significantly different between week. Different letter above the bars in each week showed significantly different at *P<0.05*.

**Figure 2.**
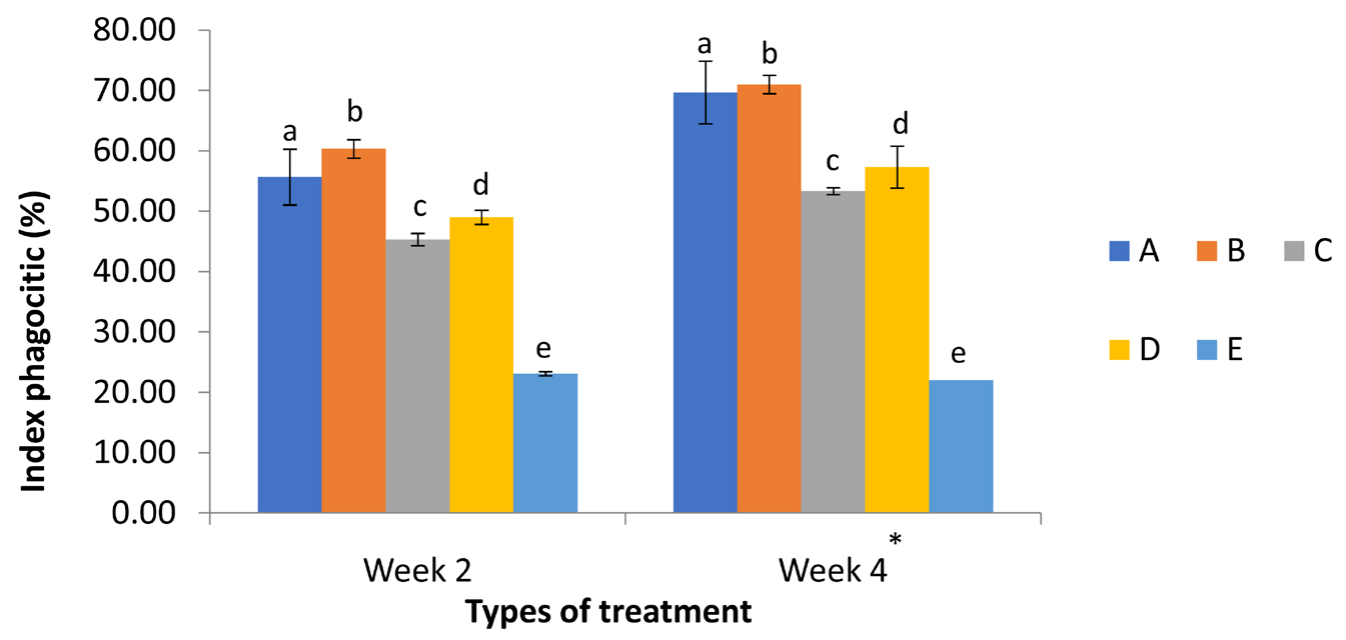
Phagocytic index (%) of Tilapia ( *Oreochromis niloticus*) fed different extract combination in prevention trials. BP = *Boesenbergia pandurata*), SF = *Solanum ferox*, and ZZ = *Zingiber Zerumbet*. In treatment trials, fish was infected with *Aeromonas hydrophila* and *Pseudomonas fluorescence.* Infected fish fed various combination and ratio of extract, namely: A = SF60/ZZ40, B = SF50/ZZ50, C = BP90/SF10, D = BP50/SF50, E = No extract addition.. Extract ratio is in mg kg ^-1^ feed. * = significantly different between week. Different letter above the bars in each week showed significantly different at *P<0.05*.

All combination extracts fed to fish in the treatment ( Figure 1) and prevention ( Figure 2) trials increased the phagocytic index. The phagocytic index of fish fed SF50/ZZ50 in the diet, in both in treatment and prevention trials, were significantly higher than control and increased from the 2 ^nd^ to 4 ^th^ week of the post-challenge test.

### Respiratory burst

**Figure 3.**
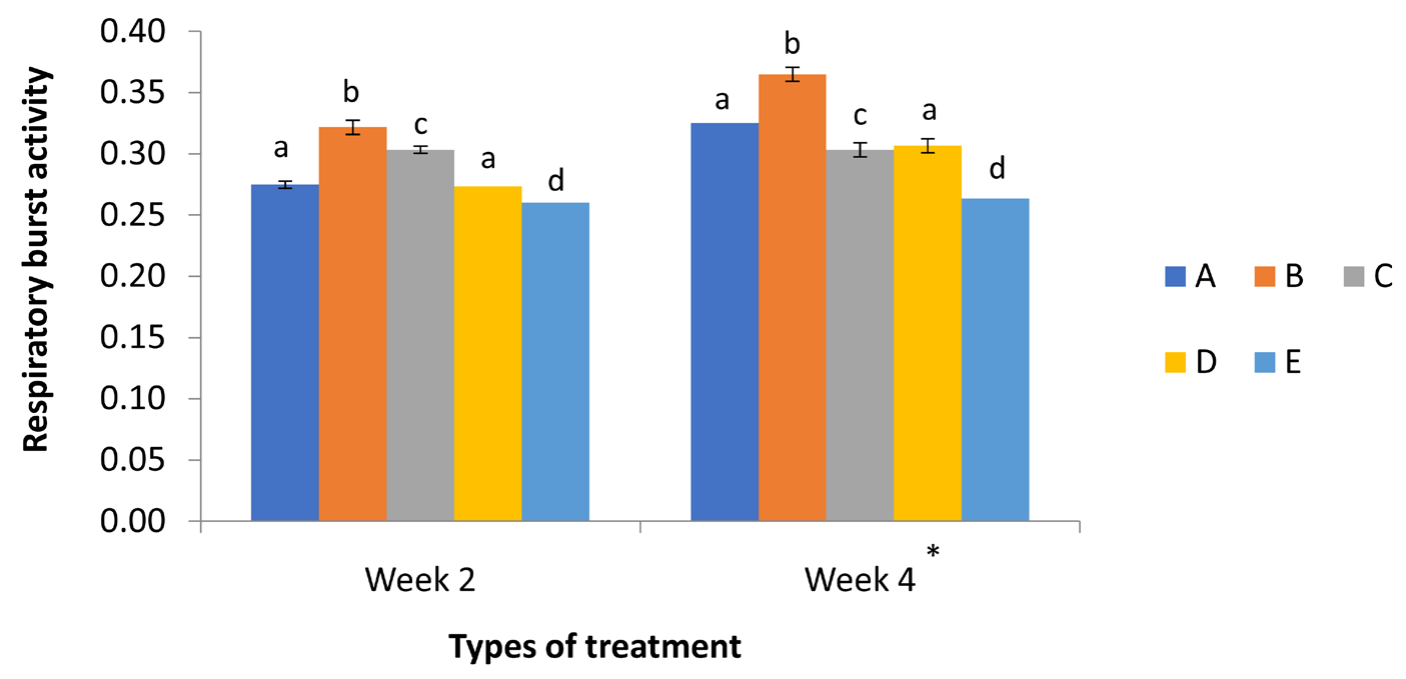
Respiratory burst activity of Tilapia ( *Oreochromis niloticus*) fed different extract combination in treatment trials. BP = *Boesenbergia pandurata*), SF = *Solanum ferox*, and ZZ = *Zingiber Zerumbet*. In treatment trials, fish was infected with *Aeromonas hydrophila* and *Pseudomonas fluorescence.* Infected fish fed various combination and ratio of extract, namely: A = SF60/ZZ40, B = SF50/ZZ50, C = BP90/SF10, D = BP50/SF50, E = No extract addition. Extract ratio is in mg kg ^-1^ feed. * = significantly different between week. Different letter above the bars in each week showed significantly different at *P<0.05*.

**Figure 4.**
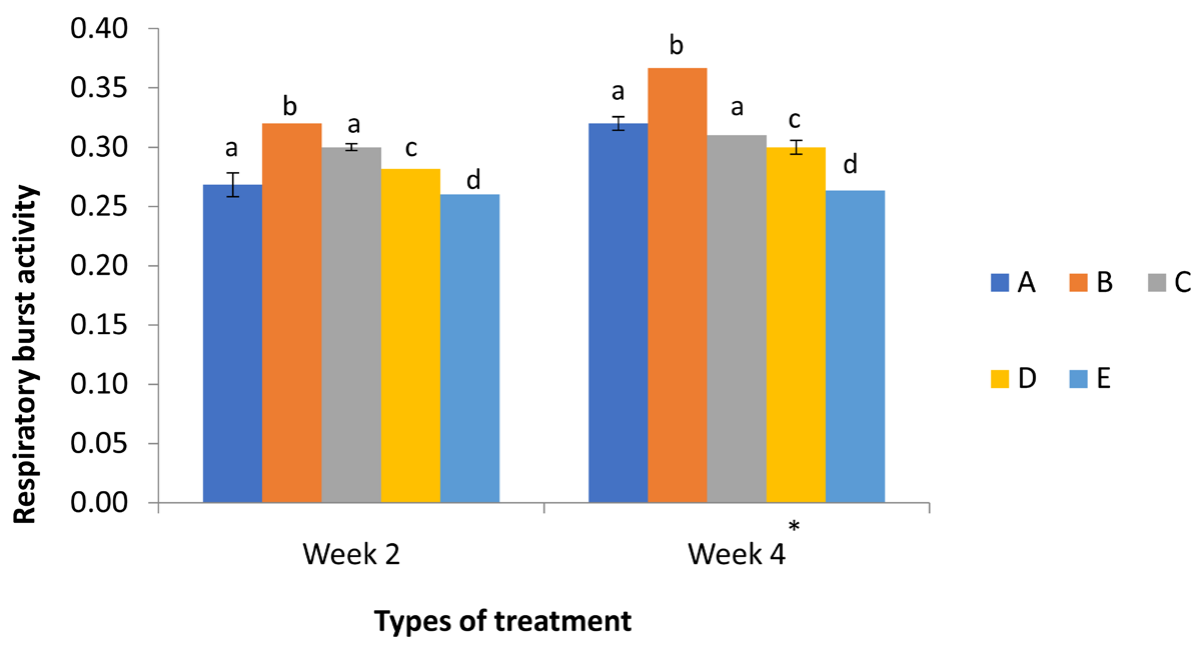
Respiratory burst activity of Tilapia ( *Oreochromis niloticus*) fed different extract combination in prevention trials. BP = *Boesenbergia pandurata*), SF = *Solanum ferox*, and ZZ = *Zingiber Zerumbet*. In treatment trials, fish was infected with *Aeromonas hydrophila* and *Pseudomonas fluorescence.* Infected fish fed various combination and ratio of extract, namely: A = SF60/ZZ40, B = SF50/ZZ50, C = BP90/SF10, D = BP50/SF50, E = No extract addition. Extract ratio is in mg kg ^-1^ feed. * = significantly different between week. Different letter above the bars in each week showed significantly different at *P<0.05*.

The respiratory burst activity of infected fish fed with combination extract increased from week 2 to week 4 in the treatment trial ( Figure 3). In addition, SF50/ZZ50 (mg per kg feed) combination extract resulted in a significantly different respiratory burst to other combinations of extracts and the control. Meanwhile, in the prevention test, infected fish fed SF50/ZZ50 combination extract in week 4 were significantly higher than control and other combinations of extracts ( *P*<0.05) ( Figure 4).

### Lysozyme activity

**Figure 5.**
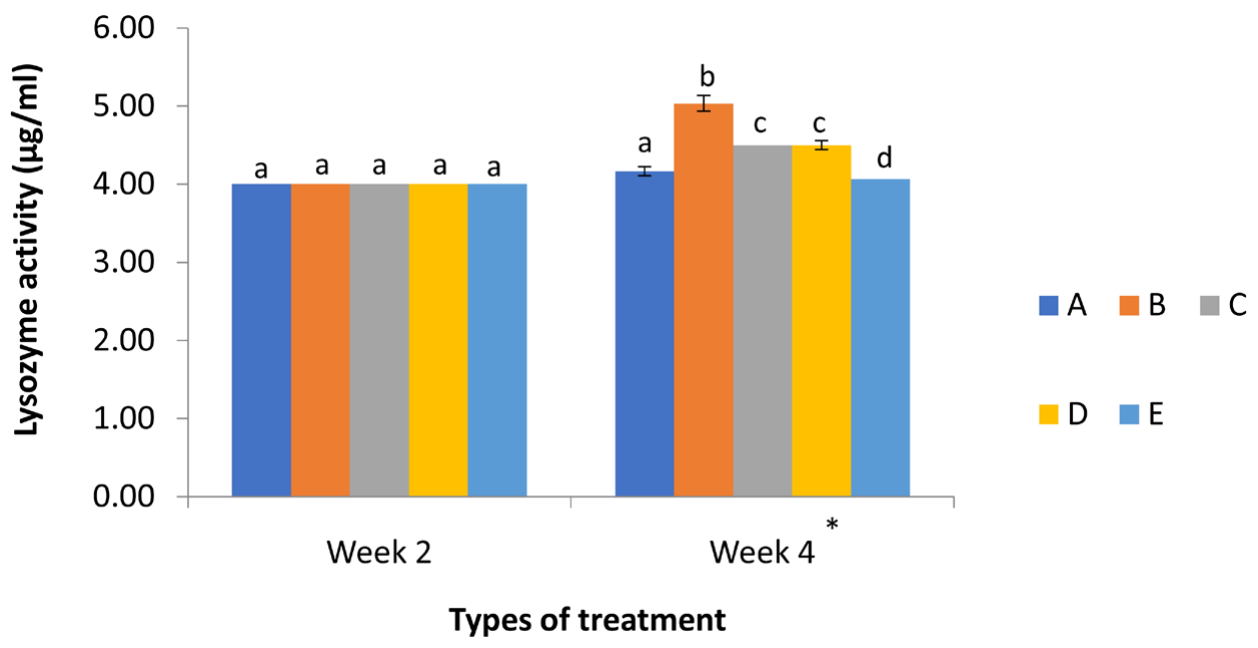
Lysozyme activity (μg mL ^-1^) of Tilapia ( *Oreochromis niloticus*) fed different extract combination in treatment trials. BP = *Boesenbergia pandurata*), SF = *Solanum ferox*, and ZZ = *Zingiber Zerumbet*. In treatment trials, fish was infected with *Aeromonas hydrophila* and *Pseudomonas fluorescence.* A = SF60/ZZ40, B = SF50/ZZ50, C = BP90/SF10, D = BP50/SF50, E = No extract addition. Extract ratio is in mg kg ^-1^ feed. * = significantly different between week. Different letter above the bars in each week showed significantly different at *P<0.05*.

**Figure 6.**
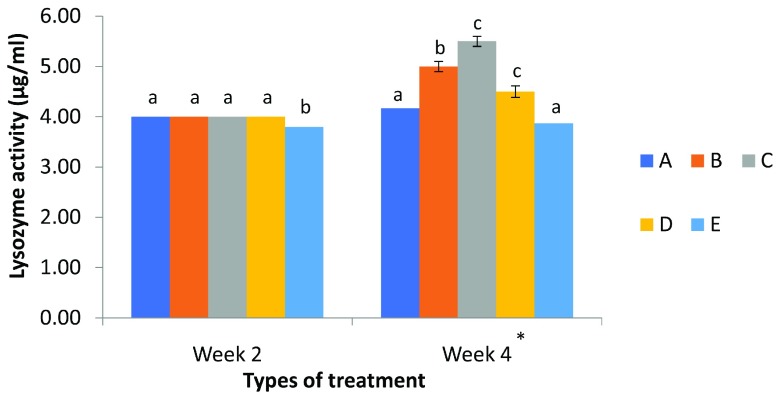
Lysozyme activity (μg mL ^-1^) of Tilapia ( *Oreochromis niloticus*) fed different extract combination in prevention trials. BP = *Boesenbergia pandurata*), SF = *Solanum ferox*, and ZZ = *Zingiber Zerumbet*. In treatment trials, fish was infected with *Aeromonas hydrophila* and *Pseudomonas fluorescence.* Infected-fish fed various combination and ratio of extract, namely: A = SF60/ZZ40, B = SF50/ZZ50, C = BP90/SF10, D = BP50/SF50, E = No extract addition. Extract ratio is in mg kg ^-1^ feed. * = significantly different between week. Different letter above the bars in each week showed significantly different at *P<0.05*.

This study revealed that lysozyme activity of infected tilapia fed SF60/ZZ40, BP90/SF10 and BP50/SF50 combinations of extract did not show a significant increase ( *P*<0.05) at weeks 2 and 4 in the treatment test. However, starting from weeks 2–4, the addition of SF50/ZZ50 combination extract in the diet of fish resulted in significantly better lysozyme activity ( Figure 5). Meanwhile, in the prevention test at weeks 2 and 4, the lysozyme activity of tilapia fed SF50/ZZ50 was significantly higher ( *P*<0.05) ( Figure 6) than in other combinations.

### TPC

The overall combination of extracts administered to treat and prevent infection by *A. hydrophila* and *P. fluorescens* may decrease the number of bacteria in the fish body until the 4 ^th^ week of observation ( Table 2). The bacterial density, in both the treatment and prevention trials was lower than in the control. Total bacteria *of A. hydrophila* and *P. fluorescens* in tilapia fish fed combination extract in the treatment trial decreased from weeks 2–4. Further, the lowest bacterial density in tilapia was obtained from the fish fed SF50/ZZ50 combination extracts in their diet, which was also significantly different ( *P*<0.05) compared to the control.

**Table 2.** Total Plate Count (10 ^6^ CFU/mL) in Tilapia ( *Oreochromis niloticus*) fed different extract combination in treatment and prevention trials.

Trials	Groups	Week	
		2	4
Treatment	A	17.4±5 ^a^	10.86±10 ^c^
	B	22.4±15 ^a^	3.05 ±10 ^d^
	C	55±10 ^b^	16±15 ^c^
	D	47±10 ^b^	4.82±10 ^d^
	E	42.85±15 ^b^	30.9±5 ^b^
Prevention	A	7.8±15 ^a^	5.71±15 ^a^
	B	9.16±5 ^b^	3.15±10 ^d^
	C	6.65±10 ^a^	4.6±5 ^d^
	D	11.15±11 ^c^	8.27±10 ^a^
	E	12.68±11 ^c^	15.3±10 ^c^

Note: Mean±standard deviation followed by different superscript letters (a,b,c,d) in the same column in each treatment or prevention trial showed significantly different at *P<0.05*. A = SF60/ZZ40, B = SF50/ZZ50, C = BP90/SF10, D = BP50/SF50, E = No extract addition. Extract ratio is in mg kg ^-1^ feed. BP = *Boesenbergia pandurata*), SF = *Solanum ferox*, and ZZ = *Zingiber Zerumbet*. CFU = Colony forming unit. In treatment and prevention trials, fish was infected with *Aeromonas hydrophila* and *Pseudomonas fluorescence.*

### Survival rate

The administration of extract with different combinations on tilapia injected with *A. hydrophila* and *P. fluorescens* bacteria increased the SR and RPS when compared to those not given the extracts ( Table 3 and Table 4). The percentage of survival of tilapia in treatment and prevention trials with combination extracts of SF 50/ZZ 50 had the highest SR compared to the other combinations of extract.

**Table 3.** Survival rate (%) of Tilapia ( *Oreochromis niloticus*) fed different extract combination in treatment and prevention trials.

Trials	Groups	Week
		4
Treatment	SF 60/ZZ 40	76.67 ^a^
	SF 50/ZZ 50	87.00 ^a^
	BP 90/SF 10	76.67,30 ^a^
	BP 50/SF 50	83.33.00 ^a^
	No extract	33.00 ^b^
Prevention	SF 60/ZZ 40	86.67 ^a^
	SF 50/ZZ 50	90 ^b^
	BP 90/SF 10	80 ^b^
	BP 50/SF 50	76.67 ^a^
	No extract	27 ^c^

Note: Mean±standard deviation followed by different superscript letters (a,b,c) in the same column in each treatment or prevention trial showed significantly different at *P<0.05*. A = SF60/ZZ40, B = SF50/ZZ50, C = BP90/SF10, D = BP50/SF50, E = No extract addition. Extract ratio is in mg per kg feed. BP = *Boesenbergia pandurata*), SF = *Solanum ferox*, and ZZ = *Zingiber Zerumbet*. In treatment and prevention trials, fish was infected with *Aeromonas hydrophila* and *Pseudomonas fluorescence.*

**Table 4.** Relative Percent Survival (RPS) of Tilapia ( *Oreochromis niloticus*) fed different extract combination in treatment and prevention trials.

Trials	Groups	Week
		4
Treatment	SF 60/ZZ 40	65 ^a^
	SF 50/ZZ 50	80 ^b^
	BP 90/SF 10	65 ^a^
	BP 50/SF 50	75 ^b^
	No extract	
Prevention	SF 60/ZZ 40	82 ^a^
	SF 50/ZZ 50	86 ^b^
	BP 90/SF 10	73 ^a^
	BP 50/SF 50	68 ^a^
	No extract	

Note: Mean±standard deviation followed by different superscript letters (a,b,c) in the same column in each treatment or prevention trial showed significantly different at *P<0.05*. A = SF60/ZZ40, B = SF50/ZZ50, C = BP90/SF10, D = BP50/SF50, E = No extract addition. Extract ratio is in ml per kg feed. BP = *Boesenbergia pandurata*), SF = *Solanum ferox*, and ZZ = *Zingiber Zerumbet*. In treatment and prevention trials, fish was infected with *Aeromonas hydrophila* and *Pseudomonas fluorescence.* Extract ratio is in mg kg ^-1^ feed.

## Discussion

The number of infectious diseases caused by pathogenic bacteria such as *A. hydrophila* have become a pivotal concern in fish culture, causing high economic losses owing to high mortality rates ^5^. The use of plant-based extracts as immunodulators has been applied to increase survival and immune system of fish to prevent or cure bacterial pathogen. Several plant extracts that contain active phytochemicals have been found and used as supplements in the feed of fish ^26– 29^.

The current study found that the WBC of tilapia infected by both bacteria in the prevention and treatment trials increased significantly ( *P*<0.05), while the RBC of tilapia infected by both bacteria in the prevention and treatment trials decreased significantly ( *P*<0.05). This result is similar to those of a previous study, which stated that the WBC increased in order to tackle the infection, while the RBC was decreased in tilapia infected with *Streptococcus agalactiae* bacteria ^30^, *S. iniae* ^10^, *A. hydrophila* and *Pseudomonas* sp. ^7^. In contrast, tilapia fed with a combination of extracts SF60/ZZ40 showed a similar RBC value both in treatment and prevention trials. In addition, tilapia fed SF50/ZZ50 in treatment trial resulted the highest RBC at the end of the trial. The Hb and Htc values were unchanged during the first week of all treatments including control; the decrease in Htc and Hb values occurred in controls without extract from weeks 2–4 post-infection in the prevention and treatment trials. This result indicated that the combined administration of the extracts was capable of improving the performance of the fish immune system by producing more WBC, thus making the fish more able to suppress the growth of bacteria in the body.

RBC, WBC, Hb and Htc can be used as an indicator of the blood profile in fish with respect to the innate immune defence and regulation of immunological function ^31^. WBC are particularly responsible for providing protection or resistance to disorders caused by infectious pathogens and non-infectious factors (nutrition, temperature and handling) ^32^. Total value of WBC also describes the health status and immune system of the fish. In addition to haematological statues, the Hb content decreases due to RBC swelling and poor Hb mobilization of the spleen and other haematopoesis organs ^33^.

Besides blood profiles, the phagocytic index, respiratory burst and lysozyme activity are good indicators for immunological status of fish during infection periods. The present results revealed that infected fish treated with a compound extract of SF50/ZZ50 showed the highest IP and increased from weeks 2–4 post-injection. These results are in line with the results of a previous study, which found that fish treated with immunostimulants usually show enhanced phagocytic cell activities ^34^. Fish have several types of phagocytic leukocytes, which are part of WBC, in the peritoneal cavity, and various tissues. The phagocytic index is also associated with the production of oxygen free radicals by using respiratory bursts, which are important events in bactericidal pathways in fish ^35, 36^.

According to Isnansetyo *et al.* ^37^, the phagocytic index is an important indicator of the enhancement of the immune system, which is showed by increasing the function of phagocytes. In addition, the increasing of phagocytic activity is affected by a complement that enhances the frequency of antigen-antibody, attaches to the receptor of phagocytosis cells, and stimulates phagocytes cells to make a forward migration to the site of infection Further, the forward migration to the site of infection has a close relationship with phagocytosis index. In addition, Secombes and Olivier ^23^ revealed that the release of superoxide anions, hydrogen peroxide and hypochlorous acid into the phagosome and extracellular space during the respiratory burst can be also considered as the pivotal mechanisms involved in the bactericidal activity of macrophages.

Total lysozyme level is a tool to measure the humoral component of the non-specific defence mechanism (innate immunity), which can be used to detect infections or injections of foreign material, including bacteria ^38– 40^. The present findings determined that tilapia fed SF 50/ZZ 50 had significantly higher ( *P*<0.05) lysozyme activity. This finding is in line with past research, stating that the lysozyme activity of Jian carp ( *Cyprinus carpio* var. Jian) ^41^ and large yellow croaker, *Pseudosciaena crocea* ^41, 42^ were increased after being fed with traditional Chinese medicine formulated from Astragalus root ( *Radix astragalin seu Heydsari*) and Chinese Angelica root ( *R. angelicae Sinenesis*).

Past research stated that the increasing survival rate and relative percent survival related to the increasing immune function of the fish which is affected by plant extract supplementation. Plant extracts containing important phytochemicals may increase monocytes, granulocytes, macrophages and neutrophils in fish, improving non-specific immune responses ^5^. Moreover, activated macrophages and neutrophils in the blood of fish also increase the number of reactive oxygen and nitrogen intermediates (ROIs and RNIs), which are toxic to bacteria ^22^.

## Conclusion

A combination of plant extracts was found to affect the health status of tilapia when compared with control. A combination of extracts of SF and ZZ (50:50 mg/kg of feed) provides the optimum protection against bacterial infections of *A. hydrophila* and *P. fluorescens* in both prevention and treatment assays.

## Data availability

Raw data for Tables and Figures can be accessed on OSF, DOI: https://doi.org/10.17605/OSF.IO/A42JB ^43^.

Data are available under the terms of the Creative Commons Zero “No rights reserved” data waiver (CC0 1.0 Public domain dedication).
