# Supplementary material for: Borneo herbal plant extracts as a natural medication for prophylaxis and treatment of Aeromonas hydrophila and Pseudomonas fluorescens infection in tilapia ( Oreochromis niloticus)
Source: F1000Res. 2019 Feb 13;7:1847. Originally published 2018 Nov 26. [Version 2] doi: 10.12688/f1000research.16902.2 (PMC6439779; doi:10.12688/f1000research.16902.2)
Supplement: Peer review history

# Peer review: Reviewer response for version 2

**DOI:** 10.5256/f1000research.19635.r44765

**Published:** 2019-03-28

**Authors:** Csaba Hancz

**Affiliations:** 1 Aquaculture and Fishery, Kaposvár University, Kaposvár, Hungary

- There is a significant weakness in the part of M&M of this article but this can be corrected after which its scientific value could surely be elevated.
- Some statistical re- evaluation of the data would also be useful.
- Please see my annotated copy of the article here which includes additional corrections and suggestions.

I have read this submission. I believe that I have an appropriate level of expertise to confirm that it is of an acceptable scientific standard, however I have significant reservations, as outlined above.

# Peer review: Reviewer response for version 2

**DOI:** 10.5256/f1000research.19635.r44431

**Published:** 2019-03-28

**Authors:** Alim Isnansetyo

**Affiliations:** 1 Department of Fisheries, Faculty of Agriculture, Gadjah Mada University, Yogyakarta, Indonesia

The authors already addressed almost all reviewer commands, and overall the manuscript quality is improved significantly. However, there is one parameter that should be clarified:

- It is still confusing between Phagocytic activity (PA) and Phagocytic Index (PI). The unit for PA is percent (%) and the unit of PI is cells or particles/Phagocyte. I think, the authors observed PA, instead of PI as the unit is %. Please replace the PI to PA.

I have read this submission. I believe that I have an appropriate level of expertise to confirm that it is of an acceptable scientific standard.

# Peer review: Reviewer response for version 2

**DOI:** 10.5256/f1000research.19635.r44432

**Published:** 2019-03-27

**Authors:** Vishnu K. Venugopal

**Affiliations:** 1 Centre for Marine Living Resources & Ecology, Kochi, Kerala , India

I am happy to note that the manuscript has now improved significantly from the original submission. The corrections made by the authors are satisfactory and I can now recommend this paper for indexing.

I have read this submission. I believe that I have an appropriate level of expertise to confirm that it is of an acceptable scientific standard.

# Peer review: Reviewer response for version 1

**DOI:** 10.5256/f1000research.18481.r41178

**Published:** 2018-12-13

**Authors:** Alim Isnansetyo

**Affiliations:** 1 Department of Fisheries, Faculty of Agriculture, Gadjah Mada University, Yogyakarta, Indonesia

This article presented the finding of Indonesian herbal extracts for preventing and treating fish diseases. This article might be indexed after several major issues are addressed:

1. Are *Boesenbergia pandurata *(BP), *Solanum ferox *(SF) and *Zingiber Zerumbet* (ZZ) typical plants in Borneo? Are the plants not found in the other parts of Indonesia? If yes, please replace the "Borneo" with "Indonesia" in the title and throughout the article.
2. Write one sentence of background in the Abstract.
3. Describe systematically in the Abstract: how to prepare the extracts, design experiment, feed preparation, infection, data collecting (hematology, non-specific immune etc.) and data analysis.
4. Write the exact concentration for the extract in mg/kg feed instead of ml/kg. Using units of ml/kg feed is not appropriate as the exact concentrations are not known. 
5. Write systematically the results in the Abstract as described in the Methods.
6. The units are written inconsistently: format (.../...., .... per ...., ....  .... ^-1^ ).
7. “Antibody titre” is a term to evaluate the effect of vaccines. To evaluate the effect of immunostimulants, we should use the term "Natural Agglutination" as we are not evaluating the specific antibody. No data are presented for Antibody titre/Natural Agglutination, even though this parameter is described in the Methods.
8. The authors are confused by the terms of phagocytic activity and phagocytic index. Phagocytic index is not described before either in the Abstract or Materials and Methods. However, the authors describe phagocytic activity in Materials and Methods. Phagocytic index and phagocytic activity are two different parameters. Please refer to some of the recommended references. Add deviation standard for each bar in all graphs.
9. Add the notation in each bar of all graphs and values in tables to show insignificant or significant difference based on DMRT test results.
10. Discussion: please interpret properly and add additional explanation about why the extracts affect the immune system of fish and increase the SR and RPS. Describe the possible constituents in the extracts by citing the previous publications.
11. Add these references in the Introduction, Materials and Methods, and Discussion: Yudiati *et al.* (2016 ^1^), Isnansetyo *et al.* (2016 ^2^) and Isnansetyo *et al.* (2015 ^3^).
12. Some grammatical errors were found.

I have read this submission. I believe that I have an appropriate level of expertise to confirm that it is of an acceptable scientific standard, however I have significant reservations, as outlined above.

## References

[1] YudiatiEIsnansetyoAMurwantokoAyuningtyasTriyantoHandayaniCR: Innate immune-stimulating and immune genes up-regulating activities of three types of alginate from Sargassum siliquosum in Pacific white shrimp, Litopenaeus vannamei. *Fish Shellfish Immunol*.2016;54: 10.1016/j.fsi.2016.03.022 46-53 10.1016/j.fsi.2016.03.022 26993614

[2] IsnansetyoAFikriyahAKasanahNMurwantoko: Non-specific immune potentiating activity of fucoidan from a tropical brown algae (Phaeophyceae), Sargassum cristaefolium in tilapia (Oreochromis niloticus). *Aquaculture International*.2016;24(2) : 10.1007/s10499-015-9938-z 465-477 10.1007/s10499-015-9938-z

[3] IsnansetyoAIrpaniHWulansariTKasanahN: Oral Administration of Alginate From A Tropical Brown Seaweed, Sargassum sp. To Enhance Non-Spesific Defense In Walking Catfish (Clarias sp.). *Aquacultura Indonesiana*.2015;15(1) : 10.21534/ai.v15i1.29 10.21534/ai.v15i1.29

# Response

**Published:** 2019-01-29

**Authors:** Rudy Nugroho

**Affiliations:** Mulawarman University, Indonesia

Dear Dr. Alim Isnansetyo,

Thank you for your valuable comment on our article. We have added some important information based on your comment and suggestions. We really appreciate it. For further details of our responses to your comment, please download the details of our responses from this link below:

https://osf.io/qwy8z/download [direct download link]

# Peer review: Reviewer response for version 1

**DOI:** 10.5256/f1000research.18481.r41177

**Published:** 2018-11-28

**Authors:** Vishnu K. Venugopal

**Affiliations:** 1 Centre for Marine Living Resources & Ecology, Kochi, Kerala , India

After checking the research article “Borneo herbal plant extracts as a natural medication for prophylaxis and treatment of Aeromonas hydrophila and Pseudomonas fluorescens infection in tilapia (Oreochromis niloticus)” by Dr. Rudy *et al.*, I reached the following suggestions to be made for the article’s acceptance:

  

1. The overall structure of the manuscript is satisfactory, though some changes are recommended.
2. In the introduction the mechanism of action of plant extracts and its medical importance could have been added.
3. The authors didn’t mention the composition of feed.
4. There is a possibility of residual ethanol in the sample. How can you conclude the results with this concern?
5. The nature and source of chemicals (Materials) used in this experiment are not mentioned.
6. Footnotes can be much clearer and the legends used in the figure should be mentioned properly. Also, in some graphs standard deviation is missing.
7. Give enough information about the figures in figure legends.
8. The Discussion part can be much stronger.

 

 In conclusion, the content of the manuscript has value for indexing. The mentioned suggestions can be considered and resubmitted.

I have read this submission. I believe that I have an appropriate level of expertise to confirm that it is of an acceptable scientific standard, however I have significant reservations, as outlined above.

## References

[1] ZahranEAbd El-GawadEARishaE: Dietary Withania sominefera root confers protective and immunotherapeutic effects against Aeromonas hydrophila infection in Nile tilapia (Oreochromis niloticus). *Fish Shellfish Immunol*.2018;80: 10.1016/j.fsi.2018.06.009 641-650 10.1016/j.fsi.2018.06.009 29886140

# Response

**Published:** 2019-01-29

**Authors:** Rudy Nugroho

**Affiliations:** Mulawarman University, Indonesia

Dear Dr. Vishnu Venugopal,

First of all, we would like to say thank you for your valuable review and comment. We have revised our article according to your review. We have also made some responses to your review - for the details of our responses, please see the link below:

http://osf.io/vzsqe/download [direct download link]
